# Human Excretion of Polybrominated Diphenyl Ether Flame Retardants: Blood, Urine, and Sweat Study

**DOI:** 10.1155/2017/3676089

**Published:** 2017-03-08

**Authors:** Shelagh K. Genuis, Detlef Birkholz, Stephen J. Genuis

**Affiliations:** ^1^Department of Emergency Medicine, Faculty of Medicine, University of Alberta, Edmonton, AB, Canada; ^2^University of Alberta, Edmonton, AB, Canada; ^3^University of Calgary, Calgary, AB, Canada

## Abstract

Commonly used as flame retardants, polybrominated diphenyl ethers (PBDEs) are routinely detected in the environment, animals, and humans. Although these persistent organic pollutants are increasingly recognized as having serious health implications, particularly for children, this is the first study, to our knowledge, to investigate an intervention for human elimination of bioaccumulated PBDEs.* Objectives.* To determine the efficacy of blood, urine, and perspiration as PBDE biomonitoring mediums; assess excretion of five common PBDE congeners (28, 47, 99, 100, and 153) in urine and perspiration; and explore the potential of induced sweating for decreasing bioaccumulated PBDEs.* Results.* PBDE congeners were not found in urine samples; findings focus on blood and perspiration. 80% of participants tested positive in one or more body fluids for PBDE 28, 100% for PBDE 47, 95% for PBDE 99, and 90% for PBDE 100 and PBDE 153. Induced perspiration facilitated excretion of the five congeners, with different rates of excretion for different congeners.* Conclusion.* Blood testing provides only a partial understanding of human PBDE bioaccumulation; testing of both blood and perspiration provides a better understanding. This study provides important baseline evidence for regular induced perspiration as a potential means for therapeutic PBDE elimination. Fetotoxic and reproductive effects of PBDE exposure highlight the importance of further detoxification research.

## 1. Introduction

Used since the early 1960s as flame retardants, polybrominated diphenyl ethers (PBDEs) were first identified as global contaminants in 1987 [1]; they were found in human adipose tissue in 1990; and in 1995 the United States Environmental Protection Agency classified deca-BDEs, a commercial mixture of PBDE congeners, as possible human carcinogens. Since that time, PBDEs have been increasingly recognized as having serious health implications for humans, particularly for children [2–6]. Comprised of a family of 209 congeners, these persistent organic pollutants [7] have been used in a wide range of everyday consumer products including polyurethane foam, textiles, plastics, electrical equipment, computers, and construction materials. Because they are not fixed in polymer matrices, PBDEs can leak over time into the surrounding environment and be dispersed [6, 8, 9]. Consequently, these lipophilic [10–13] and bioaccumulating [10, 14–16] pollutants have been routinely detected in air, soil, sewage sludge, fish, wildlife, and humans [10, 12, 17–26].

Although researchers have brought attention to the ubiquitous presence of and human health risks from PBDES, research into the elimination of bioaccumulated PBDEs has been limited to animal studies and to depuration occurring during human breastfeeding. This investigation reports the results of a study examining the concentration of five common PBDE congeners (28, 47, 99, 100, and 153) in the blood serum, urine, and perspiration of 20 study participants. The objectives of the study are to determine the efficacy of these body fluids as PBDE biomonitoring mediums, to assess the excretion of the identified congeners in urine and perspiration, and to explore the potential of induced perspiration as a means of decreasing bioaccumulated PBDEs. Data for this investigation are derived from the Blood, Urine, and Sweat (BUS) study. Other findings from this study have been published elsewhere [27–30].

### 1.1. Background

Because of their toxicity, resistance to degradation, and potential for bioaccumulation, regulatory agencies have begun to place limitations on the production and use of PBDEs. For example, two common commercial mixtures of PBDEs (PentaBDE and OctaBDE) have been banned for over 10 years in the European Union [31], and restrictions on the use of these and other mixtures of PBDEs are beginning to be implemented on a state-by-state basis in the United States [32]. Despite legislative progress, PBDE body burdens remain high in North America [6, 26, 33, 34]. Moreover, human and environmental exposure continues to be of concern as products containing these persistent pollutants are released into the environment during use, recycling, and waste processes [23, 35–40]. Researchers have, moreover, documented the long range transport of PBDEs [21–23, 41], with variance in dispersal distance depending on particle size, meteorological conditions, and the extent of bromination [25]. Given the increasing volume of research documenting the deleterious impact of PBDEs on human health (see, e.g., [3, 6, 42, 43]), it is important to briefly consider the primary routes of human exposure to PBDE congeners, mechanisms of harm, and depuration of these persistent pollutants.

#### 1.1.1. PBDE Exposure

Primary PBDE exposure routes for humans have been identified as indoor air and dust, diet, and breast milk and in utero transmission.

*Indoor Air and Dust*. Many studies identify indoor air and the concomitant inhalation, ingestion, or dermal absorption of PBDEs present in household dust as a primary PBDE exposure pathway for humans [23, 33, 44–49]. Higher levels of PBDEs in the indoor versus outdoor environment [50–52] are attributed to the breakdown in materials from consumer products and migration of PBDEs during product use. While household dust has been associated with a range of impacts, including serum concentrations of PBDEs in adults [46], altered hormone levels in men [48, 53], and elevated human milk concentrations [54], indoor work and/or recreational environments may also contribute to PBDE body burdens. Positive association has been documented between indoor dust in office environments and serum levels [55]; human exposure via indoor dust has been documented in occupational settings where there is extensive presence and/or use of electronic devices [56–58]; and, when compared to the general population, collegiate gymnasts experience higher exposure to PBDEs because of residue from the polyurethane foam in gym mats [59].

Toddlers and young children are at particular risk for PBDE accumulation because of exposure to and ingestion of indoor dust [23, 54, 60–63]. Toddlers are identified as being at increased risk primarily because of hand-to-mouth activities [3, 61] and their lower body weights, which compound the effects of exposure [43]. A striking case study published in* Environmental Health Perspectives*, for example, attributed the 2- to 15-fold higher levels of six major PBDE congeners in children, when compared to their parents, to household dust exposure [64].

*Diet*. There is considerable evidence for PBDE contamination of food; consequently, diet has also been identified as a primary source of human exposure [34, 47, 65, 66]. Because of their lipophilic characteristics and biomagnification in the food chain [67, 68], PBDEs are predominantly linked to foods of animal origin [65]. Many studies identify fish as the principal food-related PBDE source [17, 43, 69–72], with higher levels of these persistent pollutants being found in fatty or oily fish [73, 74]. Unsurprisingly, supplements containing fish oil, particularly oils derived from fish livers, the major detoxifying organ, have also been recognized as PBDE exposure pathways [65]. In North America, meats other than fish have also been established as primary food-related avenues for PBDE exposure [10, 43, 75, 76]. Meat, dairy products, and eggs, for example, are highlighted as important sources of PBDE for the average urban Canadian [54]. Although Ohta et al. [77] found higher PBDE levels in spinach than in various meats from a region of Japan, contamination of vegetables, with their low lipid and high water content, is commonly lower than in animal-based foods [69, 72]. The EFSA Panel on Contaminants in the Food Chain, for example, notes that “significantly lower” PBDE concentrations are found in the breast milk of women eating vegetarian diets than in the breast milk of those consuming animal-based products. In addition, analysis of dietary supplements rich in omega-3 fatty acids and of pure vegetable oil found that no PBDEs were present in the pure vegetable oil [65].

*Breast Milk and Fetal Exposure*. While indoor air and diet are the major ongoing sources of PBDE exposure in the general population, infant consumption of breast milk is the largest contributor to lifetime PBDE exposure [54]. This is particularly concerning given increasing levels of PBDEs in breast milk over the past 20 to 30 years [43]. Levels of contamination in human breast milk are associated with diet [65, 78], presence in household dust [54], and geographic location. Numerous studies, for example, demonstrate higher concentrations of PBDEs in the breast milk of women living in the United States and Canada than in breast milk from women living in other parts of the world [8, 26, 79, 80]. In addition to PBDE exposure via breast milk, there is substantial evidence for the prenatal exposure of neonates. Elevated PBDE levels have been found in cord blood samples [81, 82], as well as in placental tissue [83] and in the liver tissues of stillborn and live-born infants [75]. Although some studies suggest that lower-brominated PBDEs represent the primary congeners of concern for pregnant and nursing women [64, 79, 84], recent evidence suggests that higher-brominated PBDEs may undergo metabolic debromination and then transfer to the neonate or nursing infant as a more toxic metabolite or lower-brominated PBDE [26, 85].

The exposure of developing neonates and infants to PBDEs is particularly concerning in light of rapidly accumulating empirical evidence for the adverse and persisting impact of these pollutants [2, 82, 86–88]. Eskenazi et al., for example, found that maternal prenatal PBDE concentrations “were associated with impaired attention as measured by a continuous performance task at 5 years” [3]; and Herbstman et al. found that children with higher cord blood concentrations for PBDEs 47, 99, and 100 “scored lower on tests of mental and physical development at ages 12–48 and 72 months” [81]. Furthermore, in a recent review published in* The Lancet Neurology*, serious neurodevelopmental disabilities such as autism and attention-deficit hyperactivity disorder are attributed to the impact of industrial chemicals, including PBDEs, on the developing human brain [89]. Given the escalating pandemic of these disorders [90, 91], their lifelong impact of individuals and families, and the associated societal and economic burden [92], investigation into the therapeutic elimination of these persistent pollutants is timely.

#### 1.1.2. Mechanisms of Harm

A comprehensive discussion of the mechanisms of harm from PBDE congeners or their metabolites is outside the scope and goals of this investigation. It is valuable, however, to briefly highlight three of the mechanisms of harm suggested in the research literature: hormone dysregulation, cellular disruption, and neurotoxicity.

Studies indicate that PBDEs are “endocrine active” [93] with particular impact on thyroid function [65, 93, 94]. In vitro studies indicate a number of ways in which thyroid regulation is influenced by exposure to PBDEs or their metabolites [95, 96]. For example, PBDE metabolites have been found to bind thyroid hormone transport proteins and nuclear hormone receptors [93, 97, 98]. Studies also indicate T3 and/or T4 level alternations in response to PBDE exposure [99–101]. Although understanding of the PBDE impact on human thyroid function is still limited, the association between PBDE exposure and thyroid hormone disruption is supported by an increasing number of human studies (e.g., [53, 102–106]).

There is substantial evidence indicating that PBDEs and/or their metabolites interfere with physiological processes at a cellular level. In a systematic review, Costa et al. [94] identified numerous publications detailing oxidative stress-related damage from PBDE exposure, including DNA damage and apoptosis. Studies also suggest that these persistent organic pollutants interfere at a cellular level with signaling, particularly calcium homeostasis [65, 107–111], and cause mitochondrial damage [112–114].

In addition, increasing evidence demonstrates that neurotoxicity is a mechanism of harm from PBDEs [115–118]. PBDEs as a risk factor for developmental neurotoxicity have been clearly identified [43, 81, 87, 98, 107, 119], with impact on IQ and impaired learning behaviors in childhood being of particular concern [3, 87, 120].

Other pathophysiological mechanisms include epigenetic dysregulation [121–124], with particular note of emerging epigenetic links between PBDEs and autism [111, 125–127], and, very recently, detoxification impairment as a result of PBDE exposure [128]. PBDEs have also been associated with plaque formation in the brain [115], a pathological change linked to dementia [129].

#### 1.1.3. Elimination of PBDEs

Investigation of BPDE elimination has been largely restricted to in vitro and in vivo studies of rodents. Overall, studies indicate that rates of PBDE absorption, distribution, metabolism, and excretion are influenced by congener, gender, and species [6, 43]. Varying toxicokinetic patterns have been observed in mice for different PBDE congeners [16, 130], with excretion of higher brominated PBDEs being greater than that of lower-brominated PBDEs [130] and half-lives tending to increase with decreasing PBDE bromination [131]. Higher urinary excretion of PBDE 47 is reported in male as compared to female mice [16], and repeated exposure to this congener has resulted in higher tissue concentrations and decreased excretion [132]. Species-related variations have been noted in PBDE urinary excretion between mice and rats [133] and the excretion rate for PBDE 99 appears to differ between renal, biliary, and fecal excretion [134]. Different toxicokinetic patterns based on developmental stage have also been identified: higher levels of PBDEs have been found in the tissue of suckling rat pups when compared to their mothers [135], and developing rodents demonstrate reduced ability to excrete PBDEs when compared to adults [16]. To our knowledge, only one study has explored induced depuration of PBDEs in animal models. Meijer et al. used dietary supplementation with nonabsorbable fat to interrupt the enterohepatic circulation and enhance excretion of PBDE 47. Findings suggest that this approach may be used to decrease persistent organic pollutants such as PBDEs in the human body [136].

In the relative absence of studies exploring PBDE elimination in humans, animal investigations are an important means for advancing scientific investigation of these contaminants. It is important to note that congener elimination characteristics differ in animals and humans [65]. For example, PBDE half-lives in humans are much longer than in animal models [131, 137–139]. These differences complicate the extrapolation of data from animal models to humans. Human studies do, however, support a broad understanding that higher brominated PBDEs are more readily eliminated than are lower PBDE congeners [139]; that congener concentration varies across body fluids [140]; and that excretion rates vary for different congeners [140]. Although to our knowledge there are no published studies exploring mechanisms for the therapeutic elimination of PBDEs from the human body, investigation of depuration using serial samples of breast milk found that serum levels of PBDEs were not substantially reduced in samples after six months of breastfeeding [24].

## 2. Materials and Methods

### 2.1. Participant Recruitment

Nine males and 11 females with mean ages 44.5 ± 14.4 years and 45.6 ± 10.3 years, respectively, were recruited to participate in the study. Ethical approval was received from the Health Research Ethics Board of the University of Alberta. Ten participants were patients with diagnosed, chronic health conditions and 10 were healthy adults (Table 1). Participants with ongoing health concerns were recruited from the last author's clinical practice by invitation. Samples of convenience were used for both healthy and sick participants based on availability and willingness to participate. Each participant provided informed consent and voluntarily gave one 200 mL sample of blood, one sample of first morning urine, and one 100 mL sample of sweat.

### 2.2. Sample Collection

All blood samples were collected at one DynaLIFE Dx laboratory site in western Canada with vacutainer blood collection equipment (BD Vacutainer, Franklin Lakes, NJ 07417, USA) using 21-gauge stainless steel needles which were screwed into the “BD Vacutainer One-Use Holder” (REF 364815). The 10 mL glass vacutainer was directly inserted into the holder and into the back end of the needle. This process and the use of glass blood collection tubes were used to prevent contamination. Blood was collected directly into plain 10 mL glass vacutainer tubes, was allowed to clot, and after 30 minutes was centrifuged for 10 minutes at 2,000 revolutions per minute (RPM). After the serum was separated, samples were picked up by ALS Laboratories (about 3 kilometres from the blood collection site) for storage pending analysis. After being received at the ALS, serum samples were transferred to 4 mL glass vials and stored in a freezer at −20°C, pending transfer to the ALS analytical laboratory.

For urine collection, participants were instructed to collect a first morning midstream urine sample directly into a provided 500 mL glass jar container with Teflon-lined lid on the same day that blood samples were collected. Urine samples were delivered by the participants directly to ALS Laboratories. Samples were transferred by laboratory staff to 4 mL glass vials and stored in a freezer at −20°C, pending transfer.

Participants were instructed to collect perspiration from any site on their body directly into the provided 500 mL glass jar container with Teflon-lined lid. Participants washed with soap and water and rinsed their skin thoroughly before collecting perspiration. Collection was accomplished by placing the jar against their prewashed area when actively sweating or by using a stainless steel spatula against their skin to transfer perspiration directly into the glass jar. Stainless steel, a compound primarily composed of iron, chromium, and nickel, was chosen as it is the same material as the needles used in standard blood collections and does not off-gas or leach at room or body temperature. An excess of 100 mL of sweat was provided in all but one case. Each of the glass bottles used for sampling in this study was provided by ALS Laboratories and had undergone extensive cleaning and rinsing to ensure negligible risk of contamination: laboratory-grade phosphate-free detergent wash, acid rinse, hot and cold deionized water rinses, oven drying, and capping and packing in quality-controlled conditions. Sweat was collected within one week (either before or after) of collecting the blood and urine samples. Collection of perspiration occurred at any point during sweating episodes. Ten participants collected samples inside a dry infrared sauna; 7 collected samples inside a steam sauna; and 3 collected samples during and immediately after exercise. The type of exercise and location were determined by participants. Following collection, perspiration samples were delivered by the participants directly to ALS Laboratories. Samples were transferred by laboratory staff to 4 mL glass vials and stored in a freezer at −20°C, pending analysis. No preservatives were used in the jars provided for sweat and urine collection, nor in the serum storage vials.

### 2.3. Analysis

Because of congener toxicokinetic differences, we investigated elimination of five common PBDE congeners (28, 47, 99, 100, and 153) in three body fluids: blood, urine, and perspiration. For each body fluid, an isotope dilution method was used to determine levels of each congener. In accordance with standard practice, the lipid fraction of serum was analyzed and reported. Urine and perspiration were tested in their entirety. It is recognized that perspiration contains both a water component from sweat glands and a lipid component originating from sebum (sebaceous glands in hair follicles). Unlike blood testing, testing of perspiration does not fractionate into components enabling isolated lipid testing.

*Blood Analysis*. For blood, PBDE analysis included spiking the sample with a ^13^C_12_ labelled extraction standard, specific extraction and clean-up of the sample, and high resolution gas chromatography/high resolution mass spectrometry (HRGC-HRMS) measurement after addition of the ^13^C_12_ injection standard. Part of this procedure was also fat content determination.

The blood was then weighed in an Erlenmeyer flask and spiked with an extraction standard solution containing ^13^C_12_-labeled PBDEs 28, 47, 99, 100, and 153. A solution of 5 g ammonium sulphate in 20 mL of distilled water and 50 mL of methanol were added to the sample. 40 mL of hexane/diethyl ether (2/1) was added to the sample. After 10 minutes of sonication, the mixture was shaken for 10 minutes. A hexane layer was transferred into another flask. The extraction with fresh portion of hexane/diethylether was repeated. Combined hexane extracts were dried by shaking with anhydrous Na_2_SO_4_. Dried separated hexane extract was concentrated using a Kuderna-Danish apparatus to a volume of about 1 mL. Residual solvent was evaporated in an oven at 102 ± 5°C until a constant weight of the fat was achieved. Fat content was determined gravimetrically.

For clean-up, the fat was again diluted in 5 mL of hexane and transferred into a separator funnel. This is combined with the same volume of dimethyl sulfoxide. The mixture was shaken intensively for about 0.5 minutes. The DMSO layer was removed and transferred into an Erlenmeyer flask. The extraction was repeated with fresh DMSO three times. Combined DMSO portions were diluted with at least the same volume of distilled water while being cooled with running water. Reverse extraction procedures with 3 × 5 mL of n-hexane were then carried out. Combined hexane extracts were concentrated in a Kuderna-Danish apparatus to a volume of ca. 1 mL. The final extract was spiked with injection standard containing ^13^C_12_-labeled PBDE 138. Finally, 2–4 *μ*L was injected to HRGC-HRMS.

*Sweat and Urine Analysis*. For sweat and urine, whole volume of sample of sweat/urine was spiked with extraction standard solution containing ^13^C_12_-labeled PBDEs 28, 47, 99, 100, and 153 and then extracted directly after 10 minutes by shaking using 5 mL hexane. Hexane layer was transferred into another flask. The extraction with fresh portion of 5 mL hexane was repeated twice. Combined hexane extracts were dried by shaking with anhydrous Na_2_SO_4_. Dried separated hexane extract was concentrated using Kuderna-Danish apparatus to a volume of ca. 1 mL. Then, the sample extract was cleaned.

For clean-up, the extract was precleaned by shaking with 5 mL of concentrated sulphuric acid at laboratory temperature. The precleaned extract was transferred on the top of a multilayer silicagel column and eluted with hexane. The extract was concentrated with the modified Kuderna-Danish concentrator up to 0.5–1 mL. The final extract was spiked with injection standard containing ^13^C_12_-labeled PBDE 138. Finally, 2–4 *μ*L was injected to HRGC-HRMS.

*HRGC-HRMS Measurement*. Gas chromatography was performed with the following equipment: Trace GC Ultra equipped with autosampler Thermo Electron Corp. Tri Plus, column STX-500 (10 m × 0.25 mm, film 0.15 *µ*m) Restek. Mass spectrometry was performed with the following equipment: Thermo Electron Corp. DFS operated in MID mode, reference gas PFTBA, resolution *R*_10_ ≈ 10,000.

## 3. Results

Twenty participants provided samples of blood, urine, and perspiration for PBDE testing. Relevant participant characteristics are provided in Table 1. Samples from each medium were tested for five PBDE congeners: PBDEs 28, 47, 99, 100, and 153. Eighty percent of participants tested positive in one or more body fluids for PBDE 28; 100% tested positive for PBDE 47; 95% tested positive for PBDE 99; and 90% tested positive for PBDE 100 and for PBDE 153. None of the tested PBDE congeners were found in participants' urine samples (Table 2). Findings, therefore, focus on results derived from blood and perspiration testing.

Although average serum levels of all PBDEs other than PBDE 100 were higher in patients diagnosed with chronic illness (Table 3), this study does not investigate potential relationships between clinical illness and PBDEs. The BUS study as a whole tested for 120 chemical agents and found exposure to varying degrees in each participant [27]. Accordingly, it is not possible to attribute contamination from a single agent to a particular clinical outcome.

**Figure 1:**
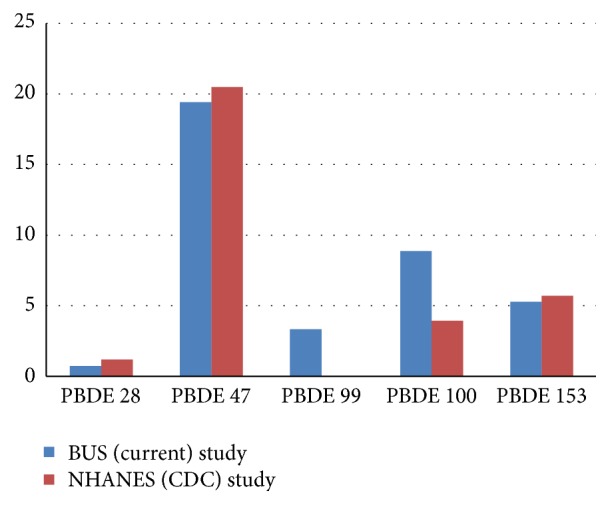
Mean blood levels for PBDE congeners in the BUS and NHANES studies [141] (results in ng/g). Geometric mean of serum concentrations (in ng/g of lipid) for the US population from the National Health and Nutrition Examination Survey.

BDEs were found in 75 of the 100 blood samples (Table 2). At least one PBDE congener was found in each of the participants' blood, with all five congeners being found in the blood of 9 participants (Table 4). PBDE 47 was present in the blood of all participants and at the highest mean concentration (Table 5). The mean amount of each PBDE congener found in blood samples from this Canadian study differed in some respects from the National Health and Nutrition Examination Survey (NHANES) conducted by the Centers for Disease Control and Prevention (CDC) [141]. Whereas the current participants had similar blood lipid levels of PBDEs 28, 47, and 153 when compared to those in the NHANES study, mean levels of PBDEs 99 and 100 were higher in the current study (Figure 1). Similar to the CDC survey where PBDE 47 was detected in nearly all participants, all participants in the current study tested positive for PBDE 47.

Because the BUS study tested for 120 chemical toxicants [27–30], there was insufficient perspiration available from three participants (participants 6, 19, and 20) to complete PBDE testing on their perspiration samples. Results for perspiration are therefore based on testing samples from 17 participants, with the end result that that there were 85 perspiration tests in total. PBDEs were found in 84 of the perspiration samples tested (Table 6). Differences in excretion rates were evident for the tested congeners. Of the five tested, PBDEs 99 and 47 were most effectively excreted into perspiration (Table 7). Excretion rates for each congener were also observed to differ between perspiration induction interventions. In this sample, participants who induced perspiration through exercise excreted the greatest proportion of PBDE 28; those who used infrared sauna excreted the most PBDE 100; and those who used steam sauna to induce perspiration excreted the most PBDE 153 (Table 8).

Of the 25 blood tests that tested negative for PBDE congeners, 16 were positive in the corresponding perspiration tests (Table 9). We were unable to triangulate eight negative blood tests due to insufficient perspiration samples from three participants. A single participant tested negative for PBDE 28 in both blood and perspiration tests.

## 4. Discussion

Findings have implications in two important areas: biomonitoring of PBDEs and human elimination of toxicants. Concern for human bioaccumulation of PBDEs and the impact of these pollutants on human health, particularly the health of neonates and children, has already resulted in the banning of these substances in many jurisdictions. Biomonitoring and estimation of body burden, however, remain a concern, particularly in North America where levels remain high.

While blood is commonly used for testing PBDE body burdens [142–144], current findings draw attention to the importance of testing both blood and perspiration. Many participants demonstrated evidence of PBDEs in perspiration, with the absence of these same agents in serum testing (Table 9). This study therefore demonstrates that perspiration testing may be useful for comprehensive understanding of PBDE body burdens. Excretion of contaminants in human perspiration has been documented for other types of pollutants, for example, toxic metals [145] and organochlorine compounds [146]. Perhaps due to the effort required to procure perspiration versus blood samples, testing commonly overlooks this medium. Testing of body fluids, however, must take into consideration the different characteristics of contaminants. Hydrophilic substances, such as cadmium and lead, for example, are found to have higher concentration in perspiration when compared to blood [27]. PBDEs, on the other hand, are lipophilic [10–13]. Although in this study perspiration provided evidence for a greater range of PBDEs than did blood serum, testing of perspiration alone, a substance that is largely composed of water, may underestimate total PBDE body burdens.

In addition to supporting previous studies which indicate that congener concentrations vary across body fluids and excretion rates vary for different congeners [140], this study provides evidence for differing PBDE excretion rates for those using different perspiration induction intervention. Sampling did not isolate perspiration gathered from different body locations (e.g., gathered from the axilla versus the torso). Moreover, participants used a single and self-selected perspiration induction method. It is therefore possible that variations in excretion rates are related to the concentration of sebaceous glands in different body areas and/or individual physiological variations. Given the small number of participants, further investigation is required to support observations regarding perspiration induction interventions.

The first and critical line of defense when dealing with PBDEs is undoubtedly to reduce exposure through better understanding exposure pathways, strategies to limit exposure to products containing PBDEs in homes, schools, and work environments, and legislative action [47, 147]. This study, however, provides baseline evidence for induced perspiration as one potential approach for the therapeutic elimination of PBDEs. The clinical usefulness of this modality is, furthermore, supported in the medical and toxicological literature: induced perspiration to diminish the body burden of toxicants has been identified as a means to prevent [148] and overcome [149, 150] illness.

Emerging evidence for the fetotoxic and reproductive effects of PBDE exposure [151] both prenatally and via breast milk and the serious health consequences of PBDEs for infants and young children [2, 3, 81, 82, 86–89] highlights the importance of PBDE elimination in at-risk populations. While the elimination of these contaminants via perspiration appears to be modest, recent studies suggest that there are synergistic effects from the coexposure to PCBs and PBDEs [110, 152, 153]. Decreasing PBDEs may, therefore, reduce the synergistic effects of PBDEs with other persistent organic pollutants. Furthermore, while the absolute amount of each PBDE congener released into sweat may be limited according to this data, an average adult may sweat more than one liter per hour during exercise. Under thermal stress, maximal rates of sweating may be as high as two to four liters/hour [154]; and sweating rates for “acclimatized” people who regularly use saunas may be as high as two liters/hour [155]. Accordingly, regular sessions of induced perspiration should be considered cumulatively as a potential clinical modality to diminish body burdens of many xenobiotics, including PBDEs. Given the impact of women's PBDE body burdens on their own health [106] and on the body burdens and health of their offspring [156] and in light of the recent admonition from the International Federation of Gynecology and Obstetrics to “make environmental health part of health care” [156], findings from this study have important implications for health professionals, particularly for those providing preconception counselling or caring for pregnant and nursing women.

### 4.1. Limitations

There are limitations associated with study design: participants were tested for only five PBDE congeners, and the small sample size does not allow extrapolation of findings to a larger population. Methods for perspiration sample collection may also impose limitations: perspiration originating from different parts of the body may excrete different concentrations of PBDEs; excretion rates may be impacted by participant's sweating duration; and factors such as ambient temperature and humidity, diet, hydration, and/or pharmaceutical and supplement use may influence excretion of PBDEs in perspiration. It is also not possible to determine whether the measured perspiration was tainted by elements originating from sebum as well as directly from skin tissue. Although it is possible that inadvertent contamination of samples occurred, precautions were taken to minimize this risk; for example, quality controls and blanks were analyzed simultaneously. Finally, it should be noted that the study did not assess health outcomes associated with PBDE elimination.

## 5. Conclusion

This is the first study, to our knowledge, to assess mechanisms for the elimination of bioaccumulated PBDEs from the human body. Our objectives were to determine the efficacy of three body fluids, blood, urine, and perspiration, as PBDE biomonitoring mediums, to assess the excretion potential of identified congeners into urine and sweat, and to explore the potential of induced perspiration as a means to decrease bioaccumulated PBDEs. None of the five tested PBDE congeners (28, 47, 99, 100, and 153) were found in participants' urine samples, suggesting that urine testing is not useful for biomonitoring or elimination of these common congeners. In this paper, we therefore focused on the results of blood and perspiration testing.

Although blood is commonly used for testing PBDE body burdens, our findings suggest that, in isolation, this approach provides only a partial understanding of human PBDE bioaccumulation. Testing of both blood and perspiration may be important for a better understanding of PBDE accrual in the human body. Moreover, it is evident that induced perspiration facilitates excretion of the five common PBDE congeners included in the study, with different rates of excretion for different congeners. We cannot draw conclusions with respect to the rate at which induced perspiration will diminish total body PBDE burden as there is currently no means to accurately assess PBDE load in the spectrum of human tissues. Nonetheless, given the relative absence of studies exploring PBDE elimination or clinical detoxification in humans, as well as the scientific consensus about the negative impact of PBDEs on human health, this study provides important baseline evidence suggesting that regular sessions of induced perspiration may facilitate the therapeutic elimination of PBDEs. Serious concerns about the fetotoxic and reproductive effects of PBDE exposure highlight the importance of further research in this area.

## Figures and Tables

**Table 1:** Participant characteristics.

Participant	Sex	Age	Clinical diagnosis
1	M	61	Diabetes, obesity, hypertension
2	F	40	Rheumatoid arthritis
3	M	38	Addiction disorder
4	F	25	Bipolar disorder
5	F	47	Lymphoma
6	F	43	Fibromyalgia
7	F	48	Depression
8	F	40	Chronic fatigue
9	F	68	Diabetes, fatigue, obesity
10	M	49	Chronic pain, cognitive decline
11	M	53	Healthy
12	M	23	Healthy
13	M	21	Healthy
14	F	47	Healthy
15	M	53	Healthy
16	F	43	Healthy
17	F	51	Healthy
18	M	46	Healthy
19	M	57	Healthy
20	F	50	Healthy

**Table 2:** PBDE congeners detected in body fluids^*∗*^.

	BDE28	BDE47	BDE99	BDE100	BDE153
Detected in body fluids (*n* = 20)	16	20	19	18	18
Detected in blood (*n* = 20)	11	20	12	15	17
Detected in perspiration (*n* = 17^*∗*^)	16	17	17	17	17
Detected in urine (*n* = 20)	0	0	0	0	0

^*∗*^Three study participants produced insufficient perspiration for successful testing.

**Table 3:** Health status of participants and mean PBDE levels.

Health status	BDE 28		BDE 47		BDE 99		BDE 100		BDE 153	
	Bl^1^	*P* ^2^	Bl	*P*	Bl	*P*	Bl	*P*	Bl	*P*
Healthy	0.31	0.017	13.26	0.6	1.61	0.55	6.7	0.012	4.06	0.072
Chronically ill	1.43	0.017	30.65	1.17	6.67	1.48	6.37	1.62	8.73	0.16

^1^Bl: results from blood testing (ng/g of blood lipid).

^2^ *P*: results from perspiration testing (ng/g of perspiration).

**Table 4:** Blood PBDE levels by congener (ng/g of blood lipid).

Participant	BDE 28	BDE 47	BDE 99	BDE 100	BDE 153
1	4.8	110	32	25	14
2	0.98	8.4	ND	ND	3.2
3	1	18	4.2	4.2	6.1
4	2.1	24	3.2	1	4.2
5	0.48	3.5	0.3	0.3	6.9
6	ND^*∗*^	6.6	ND	ND	ND
7	0.87	13	2	2.8	1.2
8	ND	30	4.2	4.8	3.3
9	1.9	71	19	23	43
10	2.2	22	4.8	2.6	5.4
11	ND	32	9.4	5.2	8.5
12	0.98	17	2.6	2.6	6.3
13	ND	1.8	ND	ND	2.4
14	ND	4.2	ND	54	3.4
15	1.2	8.7	3	1	5.5
16	ND	31	ND	1.9	7.6
17	ND	5.8	ND	ND	2.2
18	ND	19	ND	1.9	ND
19	0.92	10	1.1	0.37	ND
20	ND	3.1	ND	ND	4.7

^*∗*^ND: none detected.

**Table 5:** Geometric mean and selected percentiles of concentrations in blood (ng/g of blood lipids).

	BDE 28	BDE 47	BDE 99	BDE 100	BDE 153
Mean	0.72	19.42	3.34	8.87	5.28
50th percentile	0.77	16.00	1.33	1.90	5.10
75th percentile	1.03	24.61	3.90	5.99	6.44
90th percentile	1.84	34.43	8.54	25.48	8.62
95th percentile	2.19	48.87	13.61	36.09	11.29

**Table 6:** Perspiration PBDE levels by congener (ng/g of perspiration).

Participant	BDE 28	BDE 47	BDE 99	BDE 100	BDE 153
1	0.05	5.2	8.1	1.6	0.84
2	0.01	0.31	0.18	0.044	0.015
3	0.006	0.3	0.3	0.053	0.029
4	0.023	0.82	0.44	0.1	0.025
5	0.022	0.79	0.47	12	0.032
6	IP^*∗*^	IP	IP	IP	IP
7	0.013	0.73	0.64	0.14	0.052
8	ND	0.044	0.046	0.0094	0.0056
9	0.031	2.3	3.1	0.6	0.38
10	0.0011	0.039	0.036	0.046	0.047
11	0.014	1.2	1.4	0.26	0.15
12	0.014	1.1	0.95	0.23	0.078
13	0.0032	0.071	0.066	0.013	0.082
14	0.0034	0.074	0.052	0.013	0.004
15	0.004	0.14	0.11	0.021	0.009
16	0.041	1.8	1.4	0.32	0.13
17	0.004	0.13	0.11	0.025	0.094
18	0.052	0.3	0.28	0.064	0.028
19	IP	IP	IP	IP	IP
20	IP	IP	IP	IP	IP

^*∗*^IP: insufficient perspiration available for testing.

**Table 7:** Geometric mean and selected percentiles of concentrations in perspiration (ng/g of perspiration).

	BDE 28	BDE 47	BDE 99	BDE 100	BDE 153
Mean	0.02	0.90	0.95	0.55	0.12
50th percentile	0.02	0.61	0.63	0.15	0.09
75th percentile	0.04	1.28	1.13	0.47	0.12
90th percentile	0.05	1.98	2.01	0.99	0.23
95th percentile	0.05	2.40	2.99	2.21	0.35

**Table 8:** Effectiveness of perspiration induction interventions for PBDE excretion.

Type of Intervention	Overall mean blood : sweat ratio				
	BDE 28	BDE 47	BDE 99	BDE 100	BDE 153
Exercise (*n* = 3)	137.2	25.5	4.8	16.9	26.9
Infrared sauna (*n* = 8)	17.9	32.1	3.25	94.6	71.8
Steam sauna (*n* = 6)	69.5	26.3	5.6	2.4	121.2

Insufficient perspiration samples are not included.

**Table 9:** Comparison: PBDE congeners detected in participants' blood and sweat.

	BDE 28	BDE 47	BDE 99	BDE 100	BDE 153
No PBDEs in blood	9	0	8	5	3
No PBDEs in blood, PBDEs in sweat	6	0	6	3	1
No PBDEs in blood, no PBDEs in sweat	1	0	0	0	0
No PBDEs in blood, insufficient perspiration^*∗*^	2	0	2	2	2

^*∗*^Three study participants produced insufficient perspiration for successful testing.

## References

[1] Jansson B., Asplund L., Olsson M. (1987). Brominated flame retardants—ubiquitous environmental pollutants?. *Chemosphere*.

[2] Gascon M., Fort M., Martínez D. (2012). Polybrominated diphenyl ethers (PBDEs) in breast milk and neuropsychological development in infants. *Environmental Health Perspectives*.

[3] Eskenazi B., Chevrier J., Rauch S. A. (2013). In utero and childhood polybrominated diphenyl ether (PBDE) exposures and neurodevelopment in the CHAMACOS study. *Environmental Health Perspectives*.

[4] Brown V. (2003). Disrupting a delicate balance: environmental effects on the thyroid. *Environmental Health Perspectives*.

[5] Lopez-Espinosa M.-J., Costa O., Vizcaino E. (2015). Prenatal exposure to polybrominated flame retardants and fetal growth in the INMA Cohort (Spain). *Environmental Science and Technology*.

[6] Lyche J. L., Rosseland C., Berge G., Polder A. (2015). Human health risk associated with brominated flame-retardants (BFRs). *Environment International*.

[7] Stockholm Convention Secretariat The POPs. http://chm.pops.int/Convention/The%20POPs/tabid/673/language/en-US/Default.aspx.

[8] Siddiqi M. A., Laessig R. H., Reed K. D. (2003). Polybrominated diphenyl ethers (PBDEs): new pollutants-old diseases. *Clinical Medicine and Research*.

[9] Hale R. C., La Guardia M. J., Harvey E., Matt Mainor T. (2002). Potential role of fire retardant-treated polyurethane foam as a source of brominated diphenyl ethers to the US environment. *Chemosphere*.

[10] Hites R. A. (2004). Polybrominated diphenyl ethers in the environment and in people: a meta-analysis of concentrations. *Environmental Science and Technology*.

[11] Li L., Xie S., Cai H., Bai X., Xue Z. (2008). Quantitative structure-property relationships for octanol-water partition coefficients of polybrominated diphenyl ethers. *Chemosphere*.

[12] Darnerud P. O., Eriksen G. S., Jóhannesson T., Larsen P. B., Viluksela M. (2001). Polybrominated diphenyl ethers: occurence, dietary exposure, and toxicology. *Environmental Health Perspectives*.

[13] Verner M.-A., Bouchard M., Fritsche E., Charbonneau M., Haddad S. (2011). In vitro neurotoxicity data in human risk assessment of polybrominated diphenyl ethers (PBDEs): overview and perspectives. *Toxicology in Vitro*.

[14] di Gangi J., Blum A., Bergman Å. (2010). San antonio statement on brominated and chlorinated flame retardants. *Environmental Health Perspectives*.

[15] Kuo Y.-M., Sepúlveda M. S., Hua I., Ochoa-Acuña H. G., Sutton T. M. (2010). Bioaccumulation and biomagnification of polybrominated diphenyl ethers in a food web of Lake Michigan. *Ecotoxicology*.

[16] Staskal D. F., Hakk H., Bauer D., Diliberto J. J., Birnbaum L. S. (2006). Toxicokinetics of polybrominated diphenyl ether congeners 47, 99, 100, and 153 in Mice. *Toxicological Sciences*.

[17] Frederiksen M., Vorkamp K., Thomsen M., Knudsen L. E. (2009). Human internal and external exposure to PBDEs—a review of levels and sources. *International Journal of Hygiene and Environmental Health*.

[18] Harrad S., De Wit C. A., Abdallah M. A.-E. (2010). Indoor contamination with hexabromocyclododecanes, polybrominated diphenyl ethers, and perfluoroalkyl compounds: an important exposure pathway for people?. *Environmental Science and Technology*.

[19] Darnerud P. O. (2003). Toxic effects of brominated flame retardants in man and in wildlife. *Environment International*.

[20] Hale R. C., Alaee M., Manchester-Neesvig J. B., Stapleton H. M., Ikonomou M. G. (2003). Polybrominated diphenyl ether flame retardants in the North American environment. *Environment International*.

[21] Wang Y., Jiang G., Lam P. K. S., Li A. (2007). Polybrominated diphenyl ether in the East Asian environment: a critical review. *Environment International*.

[22] Vives I., Grimalt J. O., Lacorte S., Guillamón M., Barceló D., Rosseland B. O. (2004). Polybromodiphenyl ether flame retardants in fish from lakes in European high mountains and greenland. *Environmental Science and Technology*.

[23] Wilford B. H., Shoeib M., Harner T., Zhu J., Jones K. C. (2005). Polybrominated diphenyl ethers in indoor dust in Ottawa, Canada: implications for sources and exposure. *Environmental Science and Technology*.

[24] Hooper K., She J., Sharp M. (2007). Depuration of polybrominated diphenyl ethers (PBDEs) and polychlorinated biphenyls (PCBs) in breast milk from California first-time mothers (primiparae). *Environmental Health Perspectives*.

[25] Strandberg B., Dodder N. G., Basu I., Hites R. A. (2001). Concentrations and spatial variations of polybrominated diphenyl ethers and other organohalogen compounds in Great Lakes air. *Environmental Science and Technology*.

[26] Park J.-S., She J., Holden A. (2011). High postnatal exposures to polybrominated diphenyl ethers (PBDEs) and polychlorinated biphenyls (PCBs) via breast milk in california: does BDE-209 transfer to breast milk?. *Environmental Science and Technology*.

[27] Genuis S. J., Birkholz D., Rodushkin I., Beesoon S. (2011). Blood, urine, and sweat (BUS) study: monitoring and elimination of bioaccumulated toxic elements. *Archives of Environmental Contamination and Toxicology*.

[28] Genuis S. J., Beesoon S., Birkholz D., Lobo R. A. (2012). Human excretion of bisphenol A: Blood, Urine, and Sweat (BUS) Study. *Journal of Environmental and Public Health*.

[29] Genuis S. J., Beesoon S., Lobo R. A., Birkholz D. (2012). Human elimination of phthalate compounds: blood, urine, and sweat (BUS) study. *The Scientific World Journal*.

[30] Genuis S. J., Beesoon S., Birkholz D. (2013). Biomonitoring and elimination of perfluorinated compounds and polychlorinated biphenyls through perspiration: blood, urine, and sweat study. *ISRN Toxicology*.

[31] Cox P., Efthymiou P. (2003). Directive 2003/11/EC of the European parliament and of the council of February 6 2003 amending for the 24th time Council Directive 76/669/EEC relating to restrictions on the marketing and use of certain dangerous substances and preparations (pentabromodiphenyl ether, octabromodiphenyl ether). *Official Journal of the European Union*.

[32] Vonderheide A. P., Mueller K. E., Meija J., Welsh G. L. (2008). Polybrominated diphenyl ethers: causes for concern and knowledge gaps regarding environmental distribution, fate and toxicity. *Science of the Total Environment*.

[33] Lorber M. (2008). Exposure of Americans to polybrominated diphenyl ethers. *Journal of Exposure Science and Environmental Epidemiology*.

[34] Domingo J. L. (2012). Polybrominated diphenyl ethers in food and human dietary exposure: a review of the recent scientific literature. *Food and Chemical Toxicology*.

[35] Birnbaum L. S., Staskal D. F. (2004). Brominated flame retardants: cause for concern?. *Environmental Health Perspectives*.

[36] De Boer J., Wester P. G., Van Der Horst A., Leonards P. E. G. (2003). Polybrominated diphenyl ethers in influents, suspended particulate matter, sediments, sewage treatment plant and effluents and biota from the Netherlands. *Environmental Pollution*.

[37] Lee S., Jang Y.-C., Kim J.-G. (2015). Static and dynamic flow analysis of PBDEs in plastics from used and end-of-life TVs and computer monitors by life cycle in Korea. *Science of the Total Environment*.

[38] Streets S. S., Henderson S. A., Stoner A. D., Carlson D. L., Simcik M. F., Swackhamer D. L. (2006). Partitioning and bioaccumulation of PBDEs and PCBs in Lake Michigan. *Environmental Science and Technology*.

[39] Guo J., Zhang R., Xu Z. (2015). PBDEs emission from waste printed wiring boards during thermal process. *Environmental Science and Technology*.

[40] Schlummer M., Gruber L., Mäurer A., Wolz G., van Eldik R. (2007). Characterisation of polymer fractions from waste electrical and electronic equipment (WEEE) and implications for waste management. *Chemosphere*.

[41] Hazrati S., Harrad S. (2005). Implications of passive sampling derived concentrations of airborne PCBs and PBDEs in urban indoor microenvironments. *Organohalogen Compound*.

[42] Talsness C. E. (2008). Overview of toxicological aspects of polybrominated diphenyl ethers: a flame-retardant additive in several consumer products. *Environmental Research*.

[43] Costa L. G., Giordano G. (2007). Developmental neurotoxicity of polybrominated diphenyl ether (PBDE) flame retardants. *NeuroToxicology*.

[44] Coelho S. D., Sousa A. C. A., Isobe T., Tanabe S., Nogueira A. J. A. (2014). Flame retardants in indoor dust—a review on the levels of polybrominated diphenyl ethers and hexabromocyclododecanes. *Current Organic Chemistry*.

[45] Sjödin A., Päpke O., McGahee E. (2008). Concentration of polybrominated diphenyl ethers (PBDEs) in household dust from various countries. *Chemosphere*.

[46] Johnson P. I., Stapleton H. M., Sjodin A., Meeker J. D. (2010). Relationships between polybrominated diphenyl ether concentrations in house dust and serum. *Environmental Science and Technology*.

[47] Johnson-Restrepo B., Kannan K. (2009). An assessment of sources and pathways of human exposure to polybrominated diphenyl ethers in the United States. *Chemosphere*.

[48] Meeker J. D., Johnson P. I., Camann D., Hauser R. (2009). Polybrominated diphenyl ether (PBDE) concentrations in house dust are related to hormone levels in men. *Science of the Total Environment*.

[49] Wu N., Herrmann T., Paepke O. (2007). Human exposure to PBDEs: associations of PBDE body burdens with food consumption and house dust concentrations. *Environmental Science and Technology*.

[50] Wilford B. H., Harner T., Zhu J., Shoeib M., Jones K. C. (2004). Passive sampling survey of polybrominated diphenyl ether flame retardants in indoor and outdoor air in Ottawa, Canada: implications for sources and exposure. *Environmental Science and Technology*.

[51] Shoeib M., Harner T., Ikonomou M., Kannan K. (2004). Indoor and outdoor air concentrations and phase partitioning of perfluoroalkyl sulfonamides and polybrominated diphenyl ethers. *Environmental Science and Technology*.

[52] Butt C. M., Diamond M. L., Truong J., Ikonomou M. G., Ter Schure A. F. H. (2004). Spatial distribution of polybrominated diphenyl ethers in Southern Ontario as measured in indoor and outdoor window organic films. *Environmental Science and Technology*.

[53] Johnson P. I., Stapleton H. M., Mukherjee B., Hauser R., Meeker J. D. (2013). Associations between brominated flame retardants in house dust and hormone levels in men. *Science of the Total Environment*.

[54] Jones-Otazo H. A., Clarke J. P., Diamond M. L. (2005). Is house dust the missing exposure pathway for PBDEs? an analysis of the urban fate and human exposure to PBDEs. *Environmental Science and Technology*.

[55] Watkins D. J., McClean M. D., Fraser A. J. (2011). Exposure to PBDEs in the office environment: evaluating the relationships between dust, handwipes, and serum. *Environmental Health Perspectives*.

[56] Besis A., Katsoyiannis A., Botsaropoulou E., Samara C. (2014). Concentrations of polybrominated diphenyl ethers (PBDEs) in central air-conditioner filter dust and relevance of non-dietary exposure in occupational indoor environments in Greece. *Environmental Pollution*.

[57] Jakobsson K., Thuresson K., Rylander L., Sjödin A., Hagmar L., Bergman Å. (2002). Exposure to polybrominated diphenyl ethers and tetrabromobisphenol A among computer technicians. *Chemosphere*.

[58] Qu W., Bi X., Sheng G. (2007). Exposure to polybrominated diphenyl ethers among workers at an electronic waste dismantling region in Guangdong, China. *Environment International*.

[59] Carignan C. C., Heiger-Bernays W., McClean M. D. (2013). Flame retardant exposure among collegiate United States gymnasts. *Environmental Science and Technology*.

[60] D'Hollander W., Roosens L., Covaci A. (2010). Brominated flame retardants and perfluorinated compounds in indoor dust from homes and offices in Flanders, Belgium. *Chemosphere*.

[61] Stapleton H. M., Eagle S., Sjödin A., Webster T. F. (2012). Serum PBDEs in a North Carolina toddler cohort: associations with handwipes, house dust, and socioeconomic variables. *Environmental Health Perspectives*.

[62] Sahlström L. M. O., Sellström U., de Wit C. A., Lignell S., Darnerud P. O. L. (2015). Estimated intakes of brominated flame retardants via diet and dust compared to internal concentrations in a Swedish mother–toddler cohort. *International Journal of Hygiene and Environmental Health*.

[63] Wang W., Zheng J., Chan C.-Y., Huang M.-J., Cheung K. C., Wong M. H. (2014). Health risk assessment of exposure to polybrominated diphenyl ethers (PBDEs) contained in residential air particulate and dust in Guangzhou and Hong Kong. *Atmospheric Environment*.

[64] Fischer D., Hooper K., Athanasiadou M., Athanassiadis I., Bergman Å. (2006). Children show highest levels of polybrominated diphenyl ethers in a California family of four: a case study. *Environmental Health Perspectives*.

[65] EFSA Panel on Contaminants in the Food Chain (CONTAM) (2011). Scientific opinion on polybrominated diphenyl ethers (PBDEs) in food. *EFSA Journal*.

[66] FAO/WHO (Food and Agriculture Organization/World Health Organization) (2006). Polybrominated dipheny ethers. *Safety Evaluation of Certain Contaminants in Food*.

[67] de Wit C. A., Herzke D., Vorkamp K. (2010). Brominated flame retardants in the Arctic environment—trends and new candidates. *Science of the Total Environment*.

[68] Sørmo E. G., Salmer M. P., Jenssen B. M. (2006). Biomagnification of polybrominated diphenyl ether and hexabromocyclododecane flame retardants in the polar bear food chain in Svalbard, Norway. *Environmental Toxicology and Chemistry*.

[69] Bocio A., Llobet J. M., Domingo J. L., Corbella J., Teixidó A., Casas C. (2003). Polybrominated diphenyl ethers (PBDEs) in foodstuffs: human exposure through the diet. *Journal of Agricultural and Food Chemistry*.

[70] Chen M. Y. Y., Tang A. S. P., Ho Y. Y., Xiao Y. (2010). Dietary exposure of secondary school students in Hong Kong to polybrominated diphenyl ethers from foods of animal origin. *Food Additives and Contaminants Part A: Chemistry, Analysis, Control, Exposure and Risk Assessment*.

[71] Voorspoels S., Covaci A., Neels H., Schepens P. (2007). Dietary PBDE intake: A mArket-basket Study in Belgium. *Environment International*.

[72] Kiviranta H., Ovaskainen M.-L., Vartiainen T. (2004). Market basket study on dietary intake of PCDD/Fs, PCBs, and PBDEs in Finland. *Environment International*.

[73] Leblanc J.-C. (2006). *CALIPSO—Fish and Seafood Consumption Study and Biomarker of Exposure to Trace Elements, Pollutants and Omega 3*.

[74] Knutsen H. K., Kvalem H. E., Thomsen C. (2008). Dietary exposure to brominated flame retardants correlates with male blood levels in a selected group of Norwegians with a wide range of seafood consumption. *Molecular Nutrition and Food Research*.

[75] Schecter A., Johnson-Welch S., Tung K. C., Harris T. R., Päpke O., Rosen R. (2007). Polybrominated diphenyl ether (PBDE) levels in livers of U.S. human fetuses and newborns. *Journal of Toxicology and Environmental Health. Part A: Current Issues*.

[76] Fraser A. J., Webster T. F., McClean M. D. (2009). Diet contributes significantly to the body burden of PBDEs in the general U.S. population. *Environmental Health Perspectives*.

[77] Ohta S., Ishizuka D., Nishimura H. (2002). Comparison of polybrominated diphenyl ethers in fish, vegetables, and meats and levels in human milk of nursing women in Japan. *Chemosphere*.

[78] Pratt I., Anderson W., Crowley D. (2013). Brominated and fluorinated organic pollutants in the breast milk of first-time Irish mothers: is there a relationship to levels in food?. *Food Additives and Contaminants—Part A Chemistry, Analysis, Control, Exposure and Risk Assessment*.

[79] Schecter A., Pavuk M., Päpke O., Ryan J. J., Birnbaum L., Rosen R. (2003). Polybrominated diphenyl ethers (PBDEs) in U.S. mothers' milk. *Environmental Health Perspectives*.

[80] Law R. J., Covaci A., Harrad S. (2014). Levels and trends of PBDEs and HBCDs in the global environment: status at the end of 2012. *Environment International*.

[81] Herbstman J. B., Sjödin A., Kurzon M. (2015). Prenatal exposure to PBDEs and neurodevelopment. *Everyday Environmental Toxins: Children's Exposure Risks*.

[82] Gascon M., Vrijheid M., Martínez D. (2011). Effects of pre and postnatal exposure to low levels of polybromodiphenyl ethers on neurodevelopment and thyroid hormone levels at 4years of age. *Environment International*.

[83] Gómara B., Herrero L., Ramos J. J. (2007). Distribution of polybrominated diphenyl ethers in human umbilical cord serum, paternal serum, maternal serum, placentas, and breast milk from madrid population, Spain. *Environmental Science and Technology*.

[84] She J., Holden A., Sharp M., Tanner M., Williams-Derry C., Hooper K. (2007). Polybrominated diphenyl ethers (PBDEs) and polychlorinated biphenyls (PCBs) in breast milk from the Pacific Northwest. *Chemosphere*.

[85] Frederiksen M., Vorkamp K., Mathiesen L., Mose T., Knudsen L. E. (2010). Placental transfer of the polybrominated diphenyl ethers BDE-47, BDE-99 and BDE-209 in a human placenta perfusion system: an experimental study. *Environmental Health: A Global Access Science Source*.

[86] Ding G., Yu J., Cui C. (2015). Association between prenatal exposure to polybrominated diphenyl ethers and young children's neurodevelopment in China. *Environmental Research*.

[87] Roze E., Meijer L., Bakker A., Van Braeckel K. N. J. A., Sauer P. J. J., Bos A. F. (2009). Prenatal exposure to organohalogens, including brominated flame retardants, influences motor, cognitive, and behavioral performance at school age. *Environmental Health Perspectives*.

[88] Chen A., Yolton K., Rauch S. A. (2014). Prenatal polybrominated diphenyl ether exposures and neurodevelopment in U.S. children through 5 years of age: the HOME study. *Environmental Health Perspectives*.

[89] Grandjean P., Landrigan P. J. (2014). Neurobehavioural effects of developmental toxicity. *The Lancet Neurology*.

[90] Centers for Disease Control and Prevention (CDC) (2005). Mental health in the United States. Prevalence of diagnosis and medication treatment for attention-deficit/hyperactivity disorder—United States, 2003. *Morbidity and Mortality Weekly Report*.

[91] Autism and Developmental Disabilities Monitoring Network Surveillance Year 2008 Principal Investigators (2012). Prevalence of autism spectrum disorders—autism and developmental disabilities monitoring network, 14 sites, United States, 2008. *Morbidity and Mortality Weekly Report. Surveillance Summaries*.

[92] Trasande L., Liu Y. (2011). Reducing the staggering costs of environmental disease in children, estimated at $76.6 billion in 2008. *Health Affairs*.

[93] Dishaw L. V., Macaulay L. J., Roberts S. C., Stapleton H. M. (2014). Exposures, mechanisms, and impacts of endocrine-active flame retardants. *Current Opinion in Pharmacology*.

[94] Costa L. G., de Laat R., Tagliaferri S., Pellacani C. (2014). A mechanistic view of polybrominated diphenyl ether (PBDE) developmental neurotoxicity. *Toxicology Letters*.

[95] Butt C. M., Wang D., Stapleton H. M. (2011). Halogenated phenolic contaminants inhibit the in vitro activity of the thyroid-regulating deiodinases in human liver. *Toxicological Sciences*.

[96] Butt C. M., Stapleton H. M. (2013). Inhibition of thyroid hormone sulfotransferase activity by brominated flame retardants and halogenated phenolics. *Chemical Research in Toxicology*.

[97] Marchesini G. R., Meimaridou A., Haasnoot W. (2008). Biosensor discovery of thyroxine transport disrupting chemicals. *Toxicology and Applied Pharmacology*.

[98] Ren X.-M., Guo L.-H. (2013). Molecular toxicology of polybrominated diphenyl ethers: nuclear hormone receptor mediated pathways. *Environmental Sciences: Processes and Impacts*.

[99] Zhou T., Taylor M. M., De Vito M. J., Crofton K. M. (2002). Developmental exposure to brominated diphenyl ethers results in thyroid hormone disruption. *Toxicological Sciences*.

[100] Abdelouahab N., Langlois M.-F., Lavoie L., Corbin F., Pasquier J.-C., Takser L. (2013). Maternal and cord-blood thyroid hormone levels and exposure to polybrominated diphenyl ethers and polychlorinated biphenyls during early pregnancy. *American Journal of Epidemiology*.

[101] Herbstman J. B., Sjödin A., Apelberg B. J. (2008). Birth delivery mode modifies the associations between prenatal polychlorinated biphenyl (PCB) and polybrominated diphenyl ether (PBDE) and neonatal thyroid hormone levels. *Environmental Health Perspectives*.

[102] Oulhote Y., Chevrier J., Bouchard M. F. (2016). Exposure to polybrominated diphenyl ethers (pbdes) and hypothyroidism in canadian women. *Journal of Clinical Endocrinology and Metabolism*.

[103] Makey C. M., McClean M. D., Braverman L. E. (2016). Polybrominated diphenyl ether exposure and thyroid function tests in North American adults. *Environmental Health Perspectives*.

[104] Turyk M. E., Persky V. W., Imm P., Knobeloch L., Chatterton R., Anderson H. A. (2008). Hormone disruption by PBDEs in adult male sport fish consumers. *Environmental Health Perspectives*.

[105] Dallaire R., Dewailly É., Pereg D., Dery S., Ayotte P. (2009). Thyroid function and plasma concentrations of polyhalogenated compounds in inuit adults. *Environmental Health Perspectives*.

[106] Chevrier J., Harley K. G., Bradman A., Gharbi M., Sjödin A., Eskenazi B. (2010). Polybrominated diphenyl ether (PBDE) flame retardants and thyroid hormone during pregnancy. *Environmental Health Perspectives*.

[107] Dingemans M. M. L., de Groot A., van Kleef R. G. D. M. (2008). Hydroxylation increases the neurotoxic potential of BDE-47 to affect exocytosis and calcium homeostasis in PC12 cells. *Environmental Health Perspectives*.

[108] Dingemans M. M. L., Heusinkveld H. J., Bergman Å., van den Berg M., Westerink R. H. S. (2010). Bromination pattern of hydroxylated metabolites of BDE-47 affects their potency to release calcium from intracellular stores in PC12 cells. *Environmental Health Perspectives*.

[109] Dingemans M. M. L., van den Berg M., Bergman Å., Westerink R. H. S. (2009). Calcium-related processes involved in the inhibition of depolarization-evoked calcium increase by hydroxylated PBDEs in PC12 cells. *Toxicological Sciences*.

[110] He P., Wang A.-G., Xia T. (2009). Mechanism of the neurotoxic effect of PBDE-47 and interaction of PBDE-47 and PCB153 in enhancing toxicity in SH-SY5Y cells. *NeuroToxicology*.

[111] Napoli E., Hung C., Wong S., Giulivi C. (2013). Toxicity of the flame-retardant BDE-49 on brain mitochondria and neuronal progenitor striatal cells enhanced by a PTEN-deficient background. *Toxicological Sciences*.

[112] Coburn C. G., Currás-Collazo M. C., Kodavanti P. R. S. (2008). In vitro effects of environmentally relevant polybrominated diphenyl ether (PBDE) congeners on calcium buffering mechanisms in rat brain. *Neurochemical Research*.

[113] Pereira L. C., Miranda L. F. C., De Souza A. O., Dorta D. J. (2014). BDE-154 induces mitochondrial permeability transition and impairs mitochondrial bioenergetics. *Journal of Toxicology and Environmental Health - Part A: Current Issues*.

[114] Pazin M., Pereira L. C., Dorta D. J. (2015). Toxicity of brominated flame retardants, BDE-47 and BDE-99 stems from impaired mitochondrial bioenergetics. *Toxicology Mechanisms and Methods*.

[115] Al-Mousa F., Michelangeli F. (2012). Some commonly used brominated flame retardants cause Ca2+-atpase inhibition, beta-amyloid peptide release and apoptosis in SH-SY5Y neuronal cells. *PLoS ONE*.

[116] Goines P. E., Ashwood P. (2013). Cytokine dysregulation in autism spectrum disorders (ASD): possible role of the environment. *Neurotoxicology and Teratology*.

[117] Hendriks H. S., Antunes Fernandes E. C., Bergman Å., van den Berg M., Westerink R. H. S. (2010). PCB-47, PBDE-47, and 6-OH-PBDE-47 differentially modulate human GABAA and *α*4*β*2 nicotinic acetylcholine receptors. *Toxicological Sciences*.

[118] Li T., Wang W., Pan Y.-W., Xu L., Xia Z. (2013). A hydroxylated metabolite of flame-retardant PBDE-47 decreases the survival, proliferation, and neuronal differentiation of primary cultured adult neural stem cells and interferes with signaling of ERK5 map kinase and neurotrophin 3. *Toxicological Sciences*.

[119] Schreiber T., Gassmann K., Götz C. (2010). Polybrominated diphenyl ethers induce developmental neurotoxicity in a human in vitro model: evidence for endocrine disruption. *Environmental Health Perspectives*.

[120] Herbstman J. B., Sjödin A., Kurzon M. (2010). Prenatal exposure to PBDEs and neurodevelopment. *Environmental Health Perspectives*.

[121] Sales L. B., Kamstra J. H., Cenijn P. H., van Rijt L. S., Hamers T., Legler J. (2013). Effects of endocrine disrupting chemicals on in vitro global DNA methylation and adipocyte differentiation. *Toxicology in Vitro*.

[122] Kamstra J. H., Hruba E., Blumberg B. (2014). Transcriptional and epigenetic mechanisms underlying enhanced in vitro adipocyte differentiation by the brominated flame retardant BDE-47. *Environmental Science and Technology*.

[123] Ta N., Li C., Fang Y. (2014). Toxicity of TDCPP and TCEP on PC12 cell: changes in CAMKII, GAP43, tubulin and NF-H gene and protein levels. *Toxicology Letters*.

[124] Woods R., Vallero R. O., Golub M. S. (2012). Long-lived epigenetic interactions between perinatal PBDE exposure and Mecp2308 mutation. *Human Molecular Genetics*.

[125] Hertz-Picciotto I., Bergman Å., Fängström B. (2011). Polybrominated diphenyl ethers in relation to autism and developmental delay: A Case-Control Study. *Environmental Health: A Global Access Science Source*.

[126] Messer A. (2010). Mini-review: polybrominated diphenyl ether (PBDE) flame retardants as potential autism risk factors. *Physiology and Behavior*.

[127] Mitchell M. M., Woods R., Chi L.-H. (2012). Levels of select PCB and PBDE congeners in human postmortem brain reveal possible environmental involvement in 15q11-q13 duplication autism spectrum disorder. *Environmental and Molecular Mutagenesis*.

[128] Nicklisch S. C., Rees S. D., McGrath A. P. (2016). Global marine pollutants inhibit P-glycoprotein: environmental levels, inhibitory effects, and cocrystal structure. *Science Advances*.

[129] Bloom G. S. (2014). Amyloid-*β* and tau: the trigger and bullet in Alzheimer disease pathogenesis. *JAMA Neurology*.

[130] Gill U., Chu I., Ryan J. J., Feeley M., Ware D. G. W. (2004). Polybrominated diphenyl ethers: human tissue levels and toxicology. *Reviews of Environmental Contamination and Toxicology*.

[131] Bakker M. I., De Winter-Sorkina R., De Mul A. (2008). Dietary intake and risk evaluation of polybrominated diphenyl ethers in the Netherlands. *Molecular Nutrition and Food Research*.

[132] Staskal D. F., Diliberto J. J., Birnbaum L. S. (2006). Impact of repeated exposure on the toxicokinetics of BDE 47 in mice. *Toxicological Sciences*.

[133] Örn U., Klasson-Wehler E. (1998). Metabolism of 2,2',4,4'-tetrabromodiphenyl ether in rat and mouse. *Xenobiotica*.

[134] Hakk H., Larsen G., Klasson-Wehler E. (2002). Tissue disposition, excretion and metabolism of 2,2′,4,4′,5-pentabromodiphenyl ether (BDE-99) in the male Sprague-Dawley rat. *Xenobiotica*.

[135] Kuriyama S. N., Wanner A., Fidalgo-Neto A. A., Talsness C. E., Koerner W., Chahoud I. (2007). Developmental exposure to low-dose PBDE-99: tissue distribution and thyroid hormone levels. *Toxicology*.

[136] Meijer L., Hafkamp A. M., Bosman W. E. (2006). Nonabsorbable dietary fat enhances disposal of 2,2′,4,4′- tetrabromodiphenyl ether in rats through interruption of enterohepatic circulation. *Journal of Agricultural and Food Chemistry*.

[137] Sjödin A., Hagmar L., Klasson-Wehler E., Kronholm-Dlab K., Jakobsson E., Bergman Å. (1999). Flame retardant exposure: polybrominated diphenyl ethers in blood from Swedish workers. *Environmental Health Perspectives*.

[138] Geyer H. J., Schramm K.-W., Darnerud P. O. (2004). Terminal elimination half-lives of the brominated flame retardants TBBPA, HBCD, and lower brominated PBDEs in humans. *Organohalogen Compound*.

[139] Thuresson K., Höglund P., Hagmar L., Sjödin A., Bergman Å., Jakobsson K. (2006). Apparent half-lives of hepta- to decabrominated diphenyl ethers in human serum as determined in occupationally exposed workers. *Environmental Health Perspectives*.

[140] Inoue K., Harada K., Takenaka K. (2006). Levels and concentration ratios of polychlorinated biphenyls and polybrominated diphenyl ethers in serum and breast milk in Japanese mothers. *Environmental Health Perspectives*.

[141] Centers for Disease Control and Prevention (2015). *Fourth National Report in Human Exposure to Environmental Chemicals, Updated Tables, (February, 2015)*.

[142] Sjödin A., Wong L.-Y., Jones R. S. (2008). Serum concentrations of polybrominated diphenyl ethers (PBDEs) and polybrominated biphenyl (PBB) in the United States Population: 2003–2004. *Environmental Science and Technology*.

[143] Schecter A., Päpke O., Kuang C. T., Joseph J., Harris T. R., Dahlgren J. (2005). Polybrominated diphenyl ether flame retardants in the U.S. population: current levels, temporal trends, and comparison with dioxins, dibenzofurans, and polychlorinated biphenyls. *Journal of Occupational and Environmental Medicine*.

[144] Lee S.-J., Ikonomou M. G., Park H., Baek S.-Y., Chang Y.-S. (2007). Polybrominated diphenyl ethers in blood from Korean incinerator workers and general population. *Chemosphere*.

[145] Hohnadel D., Sunderman F., Nechay M. W., McNeely M. D., Williams T. R. (1973). Excretion of nickel, copper, zinc and lead in sweat of healthy subjects during sauna bathing. *Clinical Chemistry*.

[146] Schnare D. W., Robinson P. C. (1986). Reduction of the human body burdens of hexachlorobenzene and polychlorinated biphenyls.. *IARC scientific publications*.

[147] Brown P., Cordner A. (2011). Lessons learned from flame retardant use and regulation could enhance future control of potentially hazardous chemicals. *Health Affairs*.

[148] Laukkanen T., Kunutsor S., Kauhanen J., Laukkanen J. A. (2017). Sauna bathing is inversely associated with dementia and Alzheimer's disease in middle-aged Finnish men. *Age and Ageing*.

[149] Ross G. H., Sternquist M. C. (2012). Methamphetamine exposure and chronic illness in police officers: significant improvement with sauna-based detoxification therapy. *Toxicology and Industrial Health*.

[150] Hannuksela M. L., Ellahham S. (2001). Benefits and risks of sauna bathing. *American Journal of Medicine*.

[151] CDC—NBP—Biomonitoring Summaries—PBDEs, CDC, 2013, https://www.cdc.gov/biomonitoring/PBDEs_BiomonitoringSummary.html

[152] Eriksson P., Fischer C., Fredriksson A. (2006). Polybrominated diphenyl ethers, a group of brominated flame retardants, can interact with polychlorinated biphenyls in enhancing developmental neurobehavioral defects. *Toxicological Sciences*.

[153] Pellacani C., Tagliaferri S., Caglieri A. (2014). Synergistic interactions between PBDEs and PCBs in human neuroblastoma cells. *Environmental Toxicology*.

[154] Greger R., Windhorst U. (2013). *Comprehensive Human Physiology: From Cellular Mechanisms to Integration*.

[155] Eisalo A., Luurila O. J. (1988). The Finnish sauna and cardiovascular diseases. *Annals of Clinical Research*.

[156] Di Renzo G. C., Conry J. A., Blake J. (2015). International Federation of Gynecology and Obstetrics opinion on reproductive health impacts of exposure to toxic environmental chemicals. *International Journal of Gynecology & Obstetrics*.

